# Astrocytes-associated research in Parkinson’s disease: an explored trends analysis

**DOI:** 10.3389/fnagi.2025.1563142

**Published:** 2025-03-27

**Authors:** Yan-Jun Chen, Ming-Rong Xie, Sheng-Qiang Zhou, Fang Liu

**Affiliations:** ^1^Graduate School of Hunan University of Chinese Medicine, Changsha, China; ^2^National TCM Master Liu Zuyi Inheritance Studio, The Affiliated Hospital of Hunan Academy of Chinese Medicine, Changsha, China; ^3^The First Clinical College of Nanjing University of Chinese Medicine, Nanjing, China

**Keywords:** Parkinson’s disease, astrocytes, alpha-synuclein, neuroinflammation, oxidative stress

## Abstract

**Background:**

Parkinson’s disease (PD) is characterized pathologically by the degeneration of dopaminergic neurons and the formation of Lewy bodies. Among the various cellular and molecular mechanisms of PD, astrocyte dysfunction is one of the causes of disease development. This study aims to explore the research hotspots, frontiers, and prospective directions regarding PD and astrocytes.

**Method:**

Relevant academic publications were searched through the Web of Science database. CiteSpace, VOSviewer, and bibliometrix were used for visualization and quantitative evaluation.

**Results:**

A total of 2,408 publications related to the study topic were included in the analysis. From 2001 to 2024, annual publications gradually increased. Activated countries were concentrated in North America, Asia, and Europe. The United States and China were the main research leaders. Nanjing Medical University was the active institution with the largest number of publications, and the University of Cambridge had the highest influence on publications. *International Journal of Molecular Sciences* was the core journal with the most publications. Dr. Hu, Gang was the most productive author, and Dr. Saarma, Mart was the most influential author. Research hotspots included astrocytes, PD, neuroinflammation, alpha-synuclein (α-Syn), microglia, oxidative stress, and neurodegeneration. In recent years, NLRP3 inflammasome, extracellular vesicles (EVs), and signaling pathway were the research topics with strong burst power.

**Conclusion:**

Collaboration among different countries, organizations, and authors has effectively promoted the rapid development of this field, and the research achievements have gradually increased. The research hotspots mainly focused on neuroinflammation, α-Syn, microglia, oxidative stress, and neurodegeneration. NLRP3 inflammasome, EVs, and signaling pathway are research directions in the future.

## 1 Introduction

Parkinson’s disease (PD) is a progressive neurodegenerative disease characterized by degeneration and death of dopaminergic neurons in the substantia nigra (Kalia and Lang, 2015). Pathologically, the formation of Lewy bodies consisting mainly of aggregated α-synuclein (α-Syn) is a hallmark of the affected brain area (Tofaris, 2022). The main symptoms of PD include motor symptoms such as resting tremor, myotonia, bradykinesia, and postural balance disorder, and non-motor symptoms such as sleep disorders, cognitive impairment, and constipation (Massano and Bhatia, 2012). Although dopamine replacement therapy can temporarily relieve motor symptoms, it does not cure the disease and is sometimes accompanied by serious side effects (Li et al., 2017). Therefore, finding new therapeutic targets and methods is of great significance for improving the quality of life of PD patients.

Astrocytes are the most widely distributed glial cells in the mammalian brain and have a variety of important physiological functions. At the level of neurotransmitter regulation, astrocytes can uptake and metabolize a variety of neurotransmitters to ensure the efficient transmission of signals between neurons (Sofroniew and Vinters, 2010). Regarding neurotrophic support, astrocytes can secrete a series of neurotrophic factors, which provide continuous nutrients for neurons’ growth and normal function (Miyazaki and Asanuma, 2020). In addition, astrocytes play a crucial role in maintaining homeostasis by regulating ion balance (Kofuji and Araque, 2021). However, astrocytes are impaired in regulating neurotransmitters and neurotrophic support under pathological conditions. Activated astrocytes release numerous inflammatory mediators, which have toxic effects on neurons (Giovannoni and Quintana, 2020).

Related studies have shown a close relationship between astrocytes and PD. Genes that play pathogenic roles in PD development, such as *LRRK2*, *SNCA*, *PARK2*, *PINK1*, and *GBA*, are highly expressed in astrocyte function (Booth et al., 2017). Astrocytes can use vesicular transport to promote the diffusion of α-Syn in the central nervous system (CNS) and exacerbate the development of PD (Prymaczok et al., 2024). Inflammatory cytokines released by astrocytes are involved in the pathogenesis of PD and aggravate neuronal damage and death (Rappold and Tieu, 2010). Abnormal calcium transport in the astrocyte network can lead to dopaminergic neuronal dysfunction and further exacerbate PD (Bancroft and Srinivasan, 2021). Exploring the role of astrocytes in PD may help elucidate the underlying mechanism of the disease and provide a strategy for new therapeutic approaches.

Bibliometrics uses statistical methods to quantitatively analyze various features of publications, which can reveal the distribution structure and change rules of literature and provide researchers with an intuitive understanding of the development trends (Ninkov et al., 2022). PD and astrocyte-related publications have gradually increased in the past two decades, but there is a lack of systematic analysis and intuitive understanding. Therefore, we use bibliometrics research methods to analyze astrocytes and PD publications, aiming to reveal hotspots, frontiers, and developing trends to gain a deeper understanding of the research topics.

## 2 Methods

### 2.1 Data retrieval

Web of Science (WoS) is a powerful tool for academic literature retrieval, which can display the citation relationships among various publications and analyze research trends (Zhang et al., 2023). Data were obtained from the WoS core collection. The search formula was ((((TS = (astrocyte)) OR TS = (astrocytes)) OR TS = (astroglia)) OR TS = (astroglial cell)) AND TS = (Parkinson’s disease). The search time was set before January 1, 2025. The language was limited to English, and publication types were limited to articles and reviews. Two researchers checked the retrieved literature and manually excluded those that were not relevant to the topic of the study. Finally, 2,408 publications related to the topic were included.

### 2.2 Data analysis

The software used for data analysis is consistent with the previous study (Chen et al., 2024). CiteSpace can transform literature data into visual graphics, reveal the knowledge structure of the research field, and identify important stages of development and academic events (Chen, 2004). VOSviewer presents the complex literature relationship in the form of graphs, clearly showing the citation relationship, topic distribution, and clustering among publications (van Eck and Waltman, 2010). Bibliometrix is a comprehensive mapping analysis tool that can conduct multi-dimensional relationship analysis of various bibliometric indicators of publications (Aria and Cuccurullo, 2017).

## 3 Results

### 3.1 Publication growth trend

**Figure 1:**
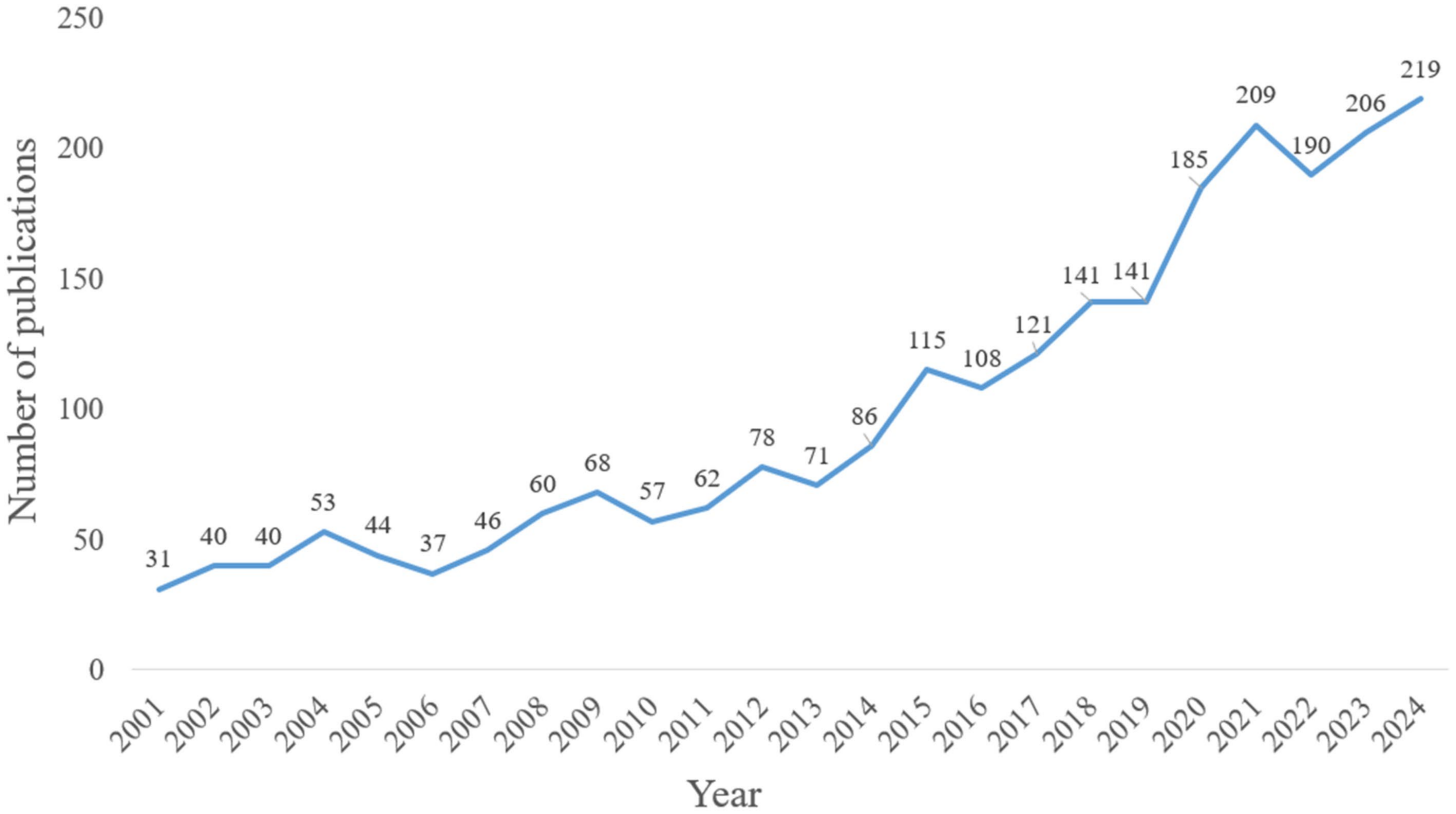
Trends in publications related to astrocytes and PD from 2001 to 2024.

From 2001 to 2024, a total of 2,408 publications on astrocytes and PD were published. The trend of publications was divided into two stages (Figure 1). In the first stage, from 2001 to 2014, the number of publications was less than 100, and the research progress was slow. In the second stage, the number of publications increased rapidly from 2015 to 2024, reaching a peak in 2024 (219 papers).

### 3.2 Geographic distribution and country

**Figure 2:**
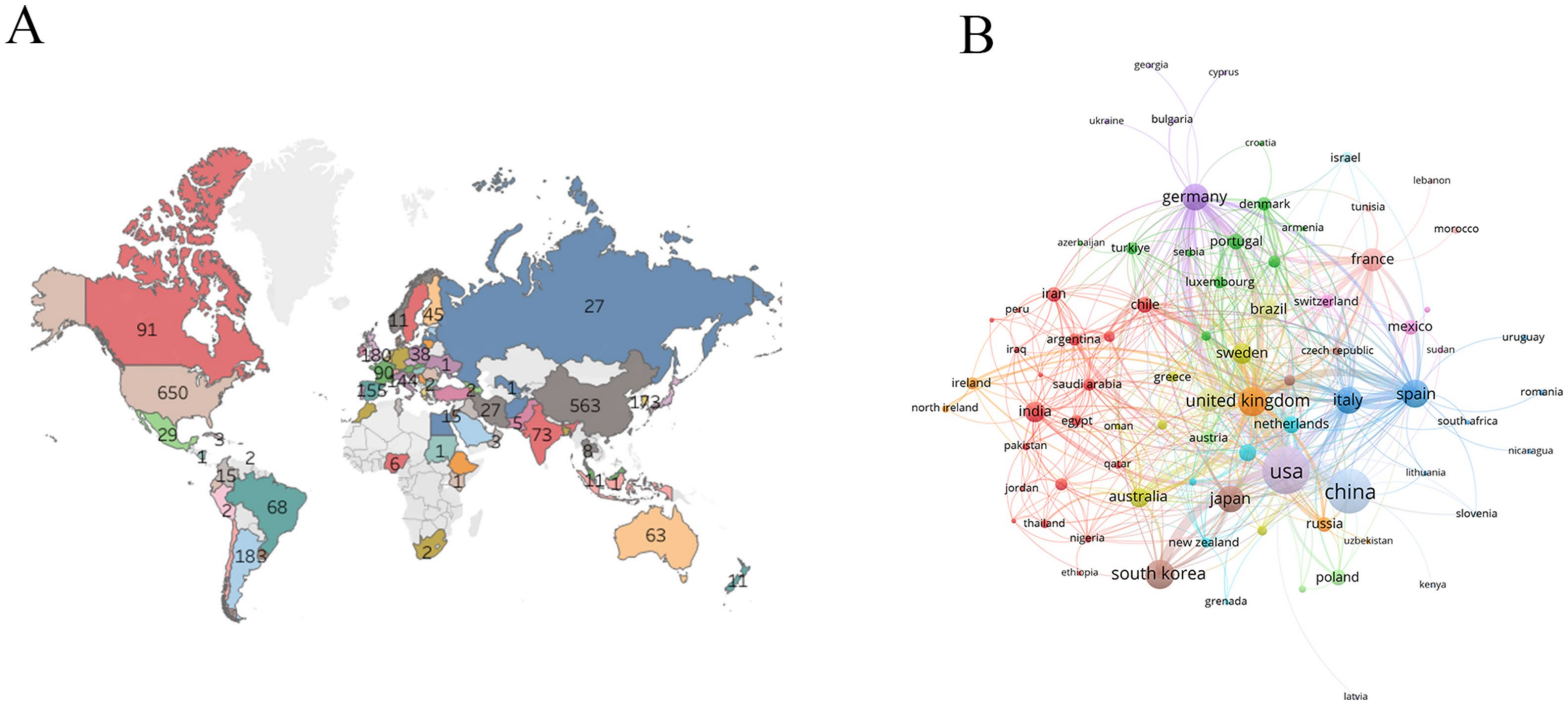
Analysis of countries. **(A)** World geographic distribution of publications. **(B)** Collaboration between countries.

The world map showed that research on astrocytes and PD was mainly concentrated in North America, Asia, and Europe (Figure 2A). The United States and China were major research forces and collaborated closely (Figure 2B). The top three countries with the highest number of publications were the United States (650 publications), China (563 publications), and the United Kingdom (180 publications) (Table 1).

**Table 1:** The top 10 productive countries.

Country	Number of publications	Citations
USA	650	46,814
China	563	17,757
The United Kingdom	180	14,899
South Korea	173	14,760
Spain	155	7,969
Germany	145	13,078
Italy	144	6,563
Japan	140	6,897
Canada	91	4,803
France	90	4,665

### 3.3 Research institutions

**Figure 3:**
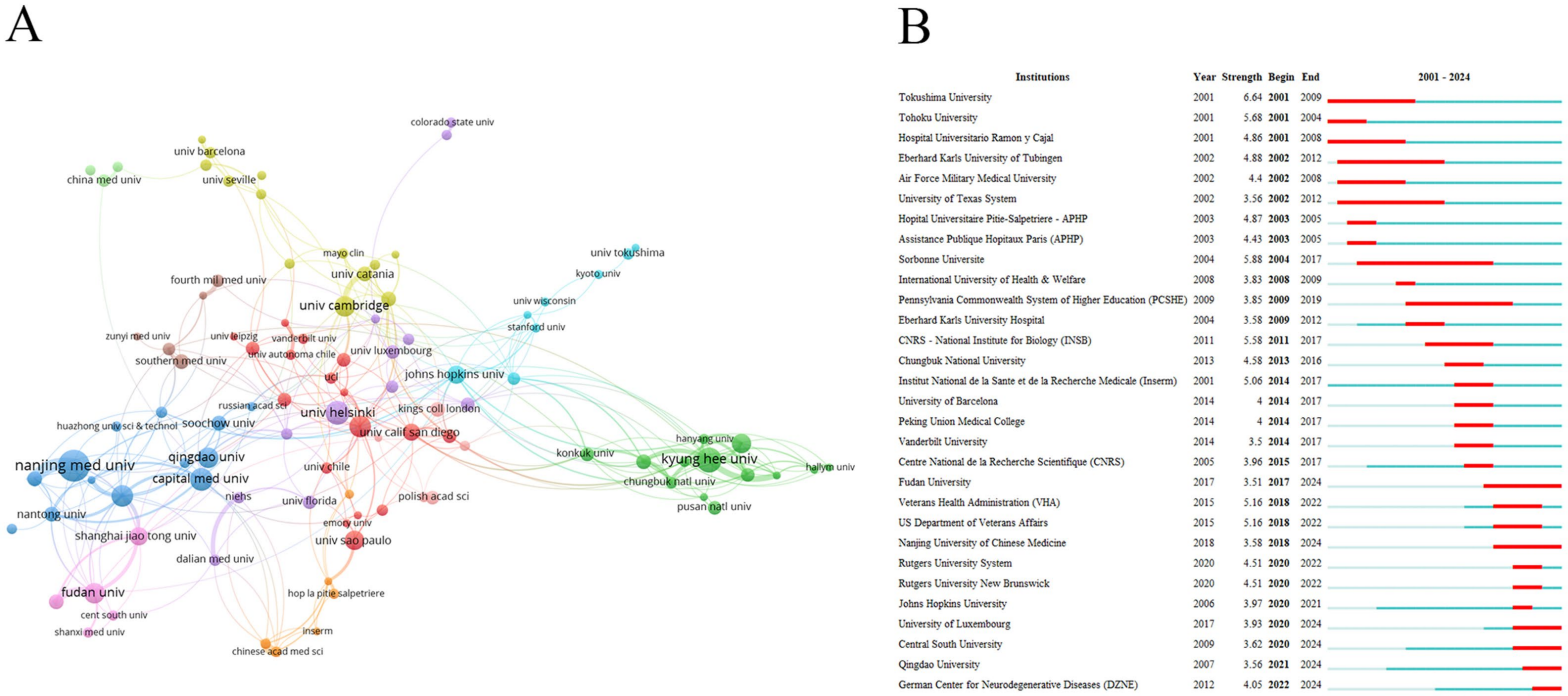
Analysis of institutions. **(A)** Collaboration between institutions. **(B)** Institutions with the strongest citation bursts.

In this research topic, Nanjing Medical University (48 publications) was the most active institution, followed by Kyung Hee University (36 publications) and the University of Helsinki (34 publications) (Table 2). Notably, the University of Cambridge has been widely recognized and concerned in the academic community, with the highest average citation (224.18 times). Institutions with a large number of publications have stable and close collaboration institutions in their respective countries (Figure 3A). Among the institutions with the strongest citation burst, Fudan University, Nanjing University of Chinese Medicine, University of Luxembourg, Central South University, Qingdao University, and the German Center for Neurodegenerative Diseases were the emerging burst forces in recent years (Figure 3B).

**Table 2:** The top 10 productive institutions.

Rank	Institution	Documents	Citations	Average number of citations
1	Nanjing Medical University	48	2,034	42.38
2	Kyung Hee University	36	1,378	38.28
3	University of Helsinki	34	2,244	66.00
4	Capital Medical University	32	806	25.19
5	Karolinska Institute	31	2,411	77.77
6	Chinese Academy of Sciences	30	1,861	62.03
7	Qingdao University	29	557	19.21
8	Fudan University	28	814	29.07
9	University of Cambridge	28	6,277	224.18
10	Ajou University	27	1,700	62.96
11	University of São Paulo	27	726	26.89

### 3.4 Journals and co-cited journals

**Figure 4:**
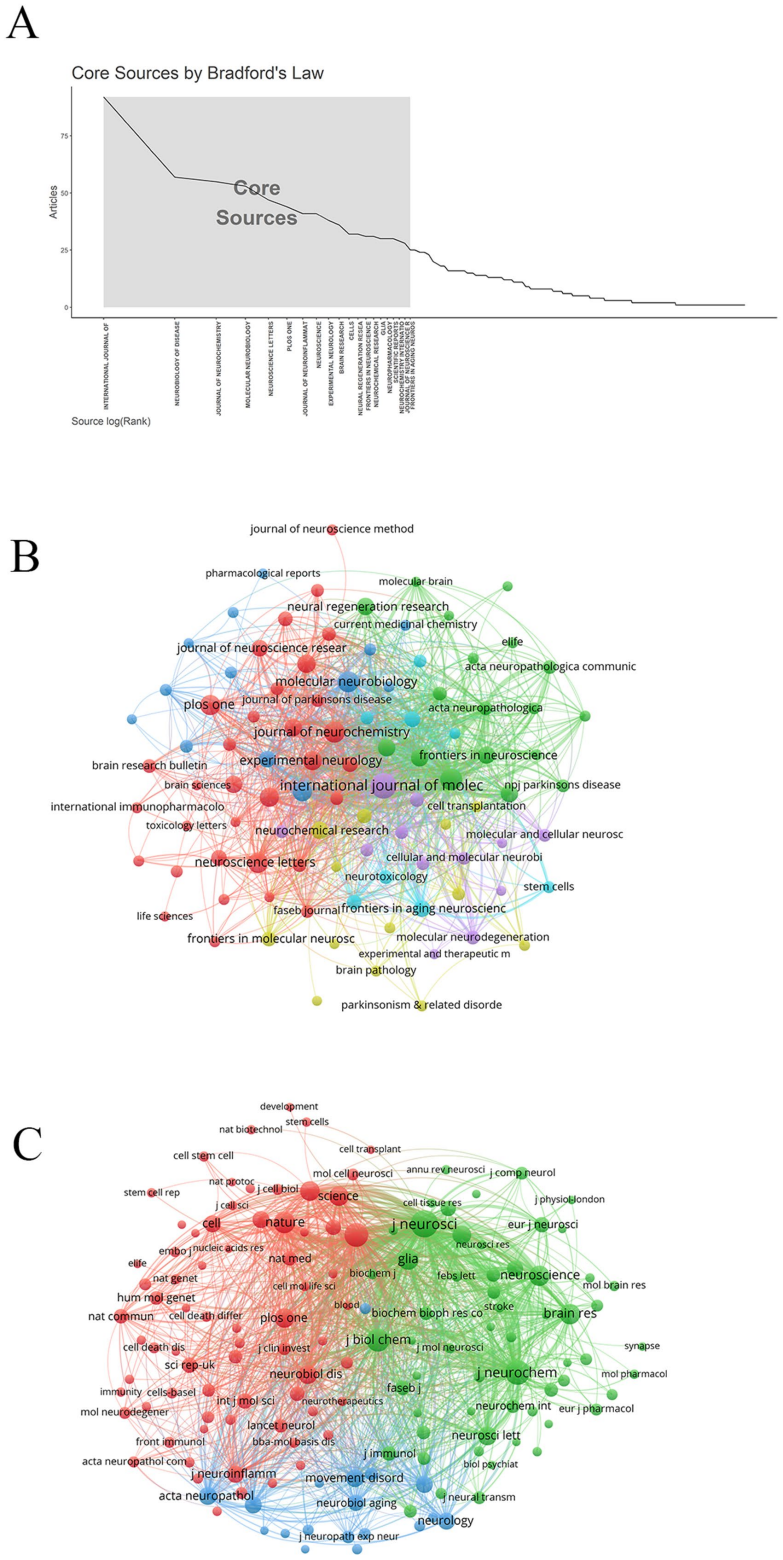
Analysis of journals and co-cited journals. **(A)** Core journal. **(B)** Journal network diagram. **(C)** Co-cited journal network diagram.

Journal analysis helps to assess journal quality and provide evidence for research decisions. According to Bradford’s Law (Bradford, 1934), 20 core journals related to research topics were identified (Figure 4A). These core journals were mainly focused on neuroscience and related fields, covering multiple aspects from basic research to clinical applications. The journal with the largest number of publications was *International Journal of Molecular Sciences* (92 publications), followed by *Neurobiology of Disease* (57 publications) and *Journal of Neurochemistry* (55 publications) (Table 3 and Figure 4B).

**Table 3:** The top 10 journals.

Rank	Source	Documents	Citations	Average number of citations	IF	JCR
1	International Journal of Molecular Sciences	92	2,255	24.51	4.9	Q1
2	Neurobiology of Disease	57	2,746	48.18	5.1	Q1
3	Journal of Neurochemistry	55	4,054	73.71	4.2	Q2
4	Molecular Neurobiology	53	2,092	39.47	4.6	Q1
5	Neuroscience Letters	47	1,409	29.98	2.5	Q3
6	PLoS One	44	1,911	43.43	2.9	Q1
7	Journal of Neuroinflammation	41	1,788	43.61	9.3	Q1
8	Neuroscience	41	1,586	38.68	2.9	Q2
9	Experimental Neurology	38	1,374	36.16	4.6	Q1
10	Brain Research	36	1,403	38.97	2.7	Q3

IF, impact factor; JCR, journal citation reports.

The analysis of co-cited journals can reveal the knowledge structure of the discipline and evaluate the mutual influence of journals. The top three co-cited journals were *Journal of Neuroscience* (6,942 citations), *Neurochemistry International* (5,315 citations), and *Proceedings of the National Academy of Sciences of the United States of America* (4,922 citations) (Table 4 and Figure 4C).

**Table 4:** The top 10 co-cited journals.

Rank	Source	Citations	IF	JCR
1	Journal of Neuroscience	6,942	4.4	Q1
2	Journal of Neurochemistry	5,315	4.2	Q2
3	Proceedings of the National Academy of Sciences of the United States of America	4,922	9.4	Q1
4	Journal of Biological Chemistry	4,175	4	Q2
5	Nature	3,539	50.5	Q1
6	Brain Research	3,241	2.7	Q3
7	Glia	3,149	5.4	Q1
8	Neuroscience	2,974	2.9	Q2
9	Science	2,822	44.8	Q1
10	Neuron	2,655	14.7	Q1

IF, impact factor; JCR, journal citation reports.

### 3.5 Authors and co-cited authors

**Figure 5:**
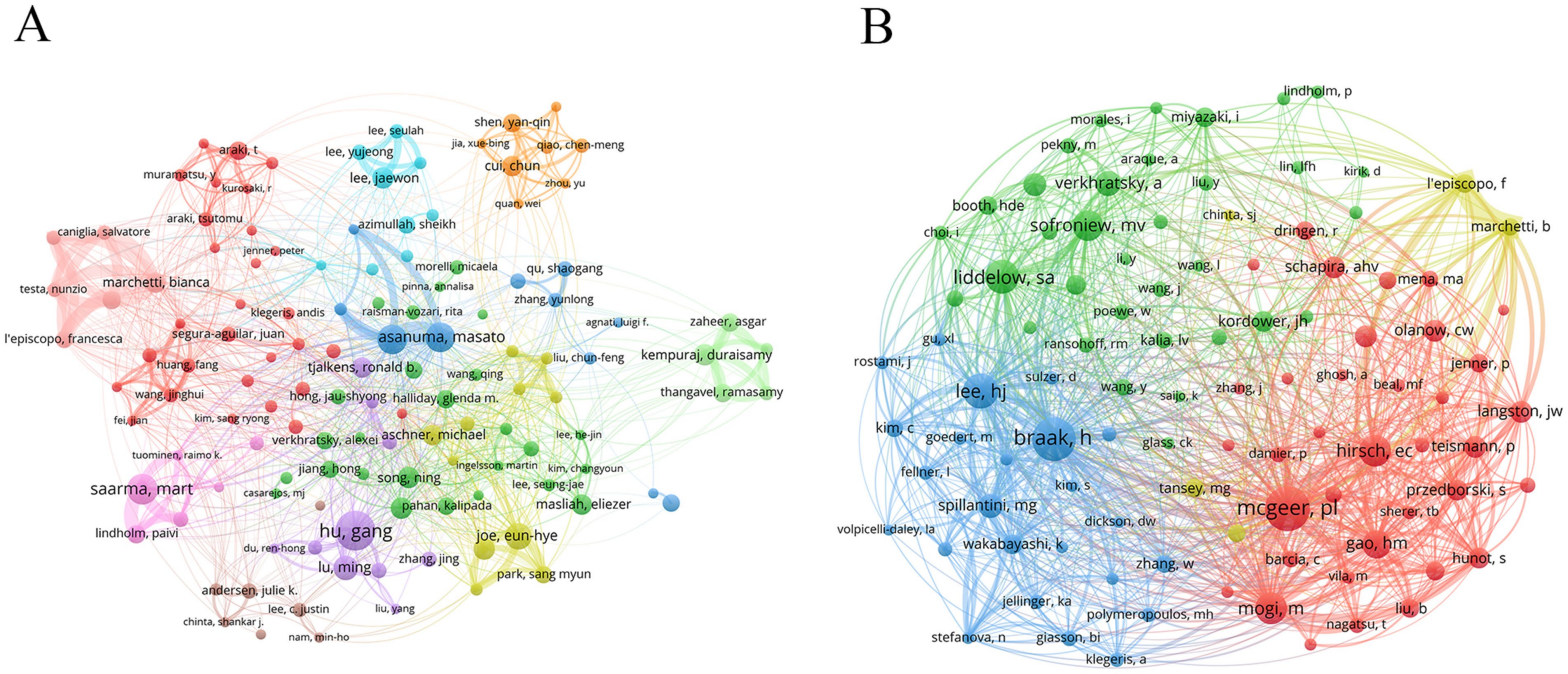
Analysis of authors and co-cited authors. **(A)** Author’s network diagram. **(B)** Co-cited author’s network diagram.

Author analysis can evaluate authors’ research output and influence, and find the core leadership in the discipline field. Dr. Hu, Gang (25 publications) was the most productive author, followed by Dr. Saarma, Mart (19 publications), Dr. Asanuma, Masato (18 publications), and Dr. Miyazaki, Ikuko (18 publications) (Table 5). Notably, Dr. Saarma, Mart was the most influential author with an average of 85.21 citations. Nodes with different colors represented different research teams, and wire lines represented collaborations (Figure 5A). Dr. Asanuma, Masato and Dr. Miyazaki, Ikuko were from the same research team and worked closely with each other.

**Table 5:** The top 10 authors.

Rank	Author	Documents	Citations	Average number of citations	Country	Institution
1	Dr. Hu, Gang	25	1,167	46.68	China	Nanjing University of Chinese Medicine
2	Dr. Saarma, Mart	19	1,619	85.21	Finland	University of Helsinki
3	Dr. Asanuma, Masato	18	789	43.83	Japan	Okayama University
4	Dr. Miyazaki, Ikuko	18	789	43.83	Japan	Okayama University
5	Dr. Joe, Eun-Hye	16	1,081	67.56	South Korea	Ajou University
6	Dr. Lu, Ming	15	788	52.53	China	Nanjing Medical University
7	Dr. Jou, Ilo	13	680	52.31	South Korea	Ajou University
8	Dr. Kempuraj Duraisamy	13	547	42.08	USA	University of Missouri Columbia
9	Dr. Lee, Jae Won	13	441	33.92	South Korea	Pusan National University
10	Dr. Song, Ning	13	310	23.85	China	Peking University
10	Dr. Xie, Junxia	13	243	18.69	China	Qingdao University

Analysis of co-cited authors can reveal disciplinary knowledge associations, find potential collaborative relationships, and track the evolution of academic ideas. The author with the highest co-citation was Dr. Mcgeer, Patrick L (531 citations), followed by Dr. Braak, Heiko (520 citations) and Dr. Lee, He-Jin (379 citations) (Table 6 and Figure 5B).

**Table 6:** The top 10 co-cited authors.

Rank	Author	Citations	Country	Institution
1	Dr. Mcgeer, Patrick L.	531	Canada	University of British Columbia
2	Dr. Braak, Heiko	520	Germany	Ulm University
3	Dr. Lee, He-Jin	379	South Korea	Konkuk University
4	Dr. Liddelow, Shane A.	364	USA	New York University School of Medicine
5	Dr. Hirsch, Etienne C.	329	France	Sorbonne University
6	Dr. Mogi, M.	327	Japan	Aichi-Gakuin University
7	Dr. Sofroniew, M. V.	308	USA	Somatix Therapy Corporation
8	Dr. Gao, Huiming	281	USA	National Institute of Environmental Health Sciences
9	Dr. Verkhratsky, Alexei	237	Lithuania	State Research Institute Centre for Innovative Medicine
10	Dr. Spillantini, M. G.	226	UK	University of Cambridge

### 3.6 Co-cited references

Co-cited reference analysis can identify the core knowledge base, research hotspots, and frontiers in the subject field. The most co-cited literature was “*Neurotoxic reactive astrocytes are induced by activated microglia*” (264 citations), followed by “*Reactive microglia are positive for HLA-DR in the substantia nigra of Parkinson’s and Alzheimer’s disease brains*” (209 citations) and “*Parkinson’s disease: Mechanisms and models*” (193 citations) (Table 7).

**Table 7:** The top 10 co-cited references.

Rank	Title	Type	Citation times	Year	Journal	IF	JCR
1	Neurotoxic reactive astrocytes are induced by activated microglia	Article	264	2017	Nature	50.5	Q1
2	Reactive microglia are positive for HLA-DR in the substantia nigra of Parkinson’s and Alzheimer’s disease brains	Article	209	1988	Neurology	8.4	Q1
3	Parkinson’s disease: mechanisms and models	Review	193	2003	Neuron	14.7	Q1
4	Direct transfer of α-synuclein from neuron to astroglia causes inflammatory responses in synucleinopathies	Article	159	2010	Journal of Biological Chemistry	4	Q2
5	Astrocytes: biology and pathology	Review	158	2010	Acta Neuropathologica	9.3	Q1
6	Staging of brain pathology related to sporadic Parkinson’s disease	Review	143	2003	Neurobiology of Aging	3.7	Q2
7	The role of astrocyte dysfunction in Parkinson’s disease pathogenesis	Review	140	2017	Trends in Neurosciences	14.6	Q1
8	Neuroinflammation in Parkinson’s disease: a target for neuroprotection?	Review	128	2009	Lancet Neurology	46.6	Q1
9	Alpha-synuclein in Lewy bodies	Article	128	1997	Nature	50.5	Q1
10	Block of A1 astrocyte conversion by microglia is neuroprotective in models of Parkinson’s disease	Article	127	2018	Nature Medicine	58.7	Q1

**Figure 6:**
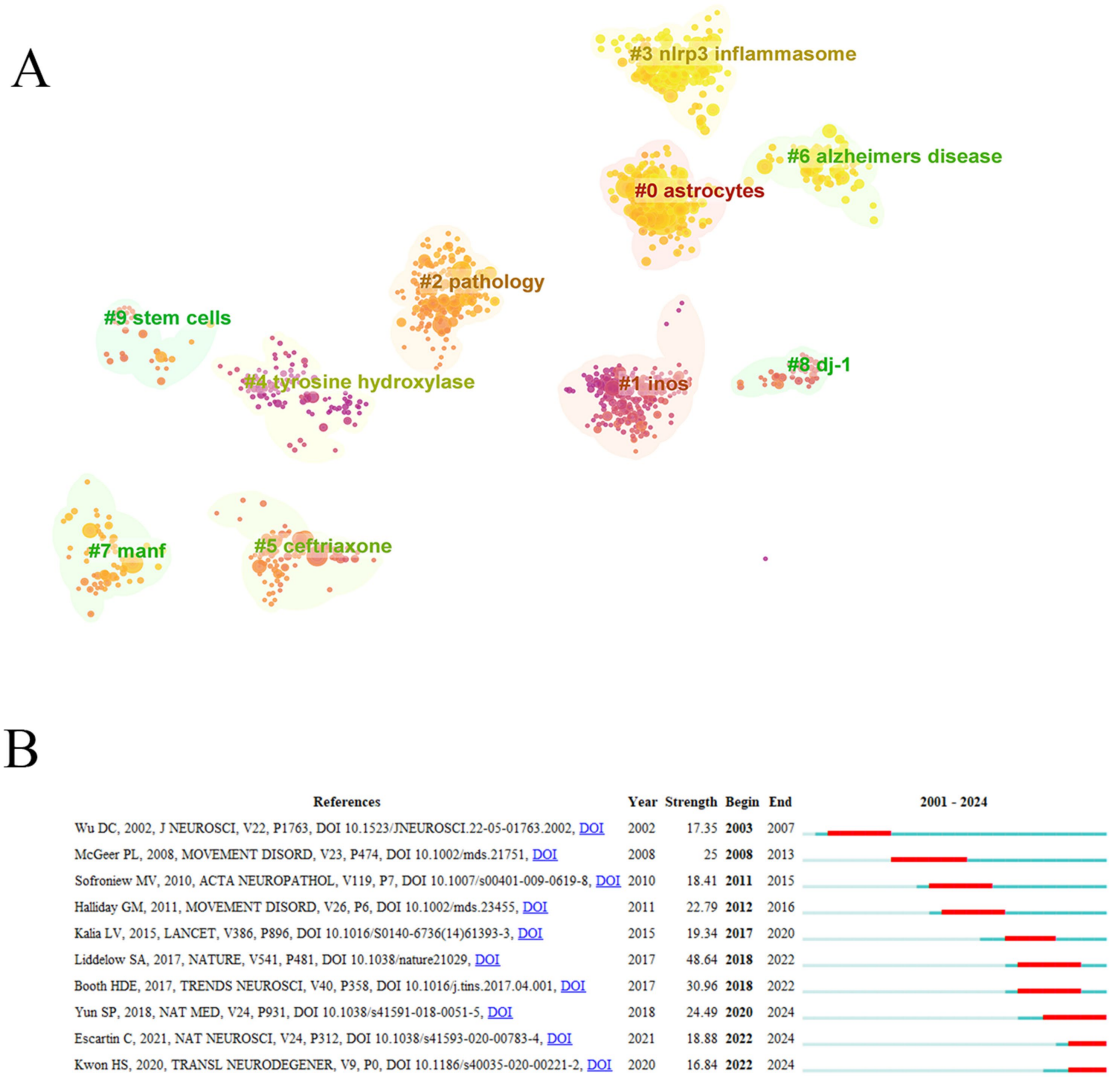
Analysis of references. **(A)** Reference cluster analysis. **(B)** References with the strongest citation bursts.

In the cluster analysis of co-cited literature, the color change from dark purple to orange to yellow reflected the evolution of research hotspots in different periods (Figure 6A). Tyrosine hydroxylase and iNOS have received much attention in the past, and the research topic has changed to NLRP3 inflammasome and pathology in recent years.

The burst of co-cited literature usually indicates that a certain research content has received high attention in a short time. “*Block of A1 astrocyte conversion by microglia is neuroprotective in models of Parkinson’s disease*” “*Reactive astrocyte nomenclature, definitions, and future directions*,” and “*Neuroinflammation in neurodegenerative disorders: the roles of microglia and astrocytes*” were references with strong burst power in recent years (Figure 6B).

### 3.7 Keywords

**Figure 7:**
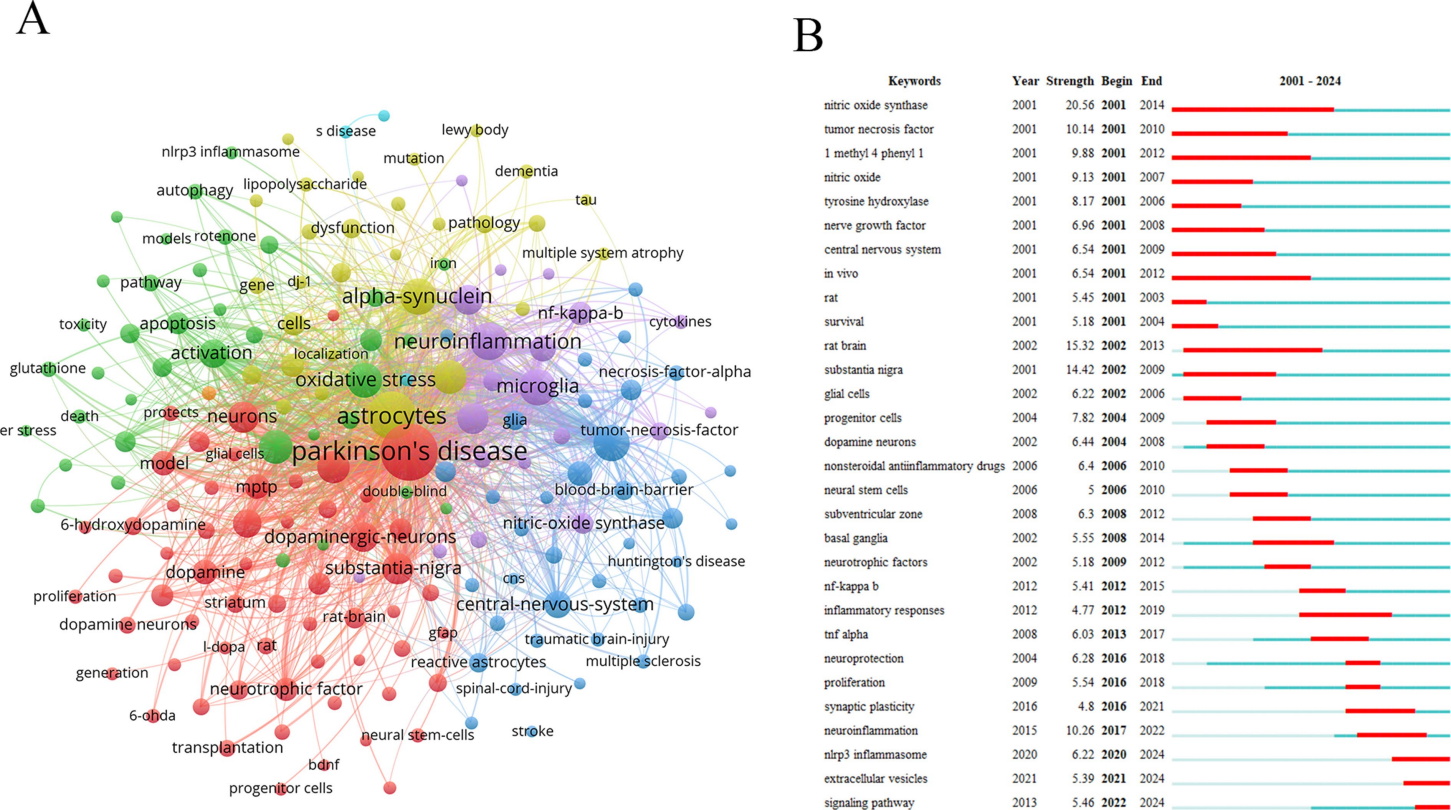
Analysis of keywords. **(A)** Keywords network diagram. **(B)** Keywords with the strongest citation bursts.

Keyword analysis can reveal the core topics of a research field, research hotspots, and the correlation between different topics. The nodes with different colors of keywords represented different cluster units (Figure 7A). The cluster of red nodes focused on animal models, cell models, and neuronal protection of PD. The cluster of green nodes focused on the mechanism of oxidative stress. The cluster of blue nodes focused on some neurodegenerative diseases, such as Alzheimer’s disease and Huntington’s disease. The cluster of yellow nodes focused on the typical pathological features of PD, including α-Syn and Lewy bodies. The purple node cluster was mainly related to inflammation. High-frequency keywords usually represent current research hotspots. In addition to astrocytes and PD, neuroinflammation, α-Syn, microglia, oxidative stress, and neurodegeneration were high-frequency keywords in the research topic.

Keyword burst refers to a sharp increase in the frequency of keywords in the literature related to a topic during a particular period. These topics have attracted the attention of many researchers in a short time, which may be emerging research directions. NLRP3 inflammasome, extracellular vesicles (EVs), and signaling pathway were the keywords with strong burst force in recent years (Figure 7B).

## 4 Discussion

Relevant studies showed astrocytes were closely related to the onset and progression of PD and played a key role in the pathophysiological mechanism of PD. This study aims to explore the research hotspots and frontier trends in the field of PD and astrocytes and to open up a new thinking path for the treatment strategy of PD.

A total of 2,408 publications related to the study topic were included in the analysis. From 2001 to 2024, annual publications gradually increased, indicating that this research topic has received increasing attention. Activated countries were concentrated in North America, Asia, and Europe. The United States and China were the main research leaders. Nanjing Medical University was the active institution with the largest number of publications, and the University of Cambridge had the highest influence on publications. Core journals were mainly focused on neuroscience and related fields, covering multiple aspects from basic research to clinical application.

Dr. Hu, Gang, Dr. Saarma, Mart, Dr. Asanuma, Masato, and Dr. Miyazaki, Ikuko were the main leaders, and their research achievements have contributed significantly to the development of the field. Dr. Hu, Gang focused on the effects of various functions of astrocytes on PD, including inflammatory response (Zhu et al., 2018), ion channel function (Hu et al., 2019), metabolic regulation (Liu et al., 2022), and signaling pathway regulation (Yin et al., 2023). In addition, he found some neuroprotective drugs. Astragaloside lV reduces dopaminergic neurodegeneration in PD by inhibiting astrocyte senescence (Xia et al., 2020). Ginkgolide decreases nerve cell damage by enhancing astrocyte clearance of α-Syn (Hua et al., 2019). Talniflumate alleviates neuroinflammation in PD by blocking the binding of aspartic acid/glutamate transporter 2 and NLRP3 inflammasome in astrocytes. Dr. Saarma, Mart focused on the structure and function of specific neuroprotective factors such as cerebral dopamine neurotrophic factor (CDNF) and mesencephalic astrocyte-derived neurotrophic factor and their therapeutic potential for PD (Lindahl et al., 2017). He also focused on current modifications used to treat PD and explored endoplasmic reticulum stress regulators as new drug targets for PD (Kovaleva and Saarma, 2021). Dr. Asanuma, Masato and Dr. Miyazaki, Ikuko were from the same research group and worked closely together. They found 5-HT_1A_ receptors (Miyazaki and Asanuma, 2016) and high mobility group box-1 (Sasaki et al., 2016) on astrocytes as potential targets for PD treatment. They also explored the dopaminergic neuroprotective effects of levetiracetam (Miyazaki et al., 2016), rotigotine (Isooka et al., 2020), and mirtazapine (Kikuoka et al., 2020) in PD by acting astrocytes. These core authors have a vital influence on academic communication, and their research direction and achievements can guide the development of the field.

High-frequency keywords intuitively reflect the topics that have received wide attention in a research field. In addition to astrocytes and PD, neuroinflammation, α-Syn, microglia, oxidative stress, and neurodegeneration were high-frequency keywords in the research topic. Astrocytes play a vital role in maintaining the integrity of the blood-brain barrier (BBB) under normal physiological conditions (Abbott et al., 2006). However, in PD, activated astrocytes lead to increased BBB permeability, and peripheral immune cells and inflammatory factors are more likely to enter the CNS (Cabezas et al., 2014). At the same time, activated astrocytes in the brain release numerous proinflammatory cytokines such as interleukin-1β and interleukin-6, which aggravate neuroinflammation (Lau and Yu, 2001). Abnormal aggregation of α-Syn can lead to lysosomal dysfunction of astrocytes and increase the release of EVs, which may exacerbate PD (Wang et al., 2023). Astrocytes can uptake and clear α-Syn. However, in the case of mutations in the PD-related gene *ATP13A2*, this protective function of astrocytes is impaired, leading to increased accumulation of α-Syn within neurons (Tsunemi et al., 2020). Activated microglia induce reactive astrocytes (A1 astrocyte) with neurotoxicity (Liddelow et al., 2017), and blockade of A1 astrocyte expression has neuroprotective effects (Yun et al., 2018). Under physiological conditions, astrocytes produce endogenous antioxidants such as glutathione and superoxide dismutase and detoxify reactive oxygen species (ROS) and reactive nitrogen species (RNS) (Stewart et al., 2002). Cellular stress and inflammation induce reactive astrogliosis, which can trigger the generation of ROS/RNS in astrocytes and may contribute to oxidative/nitrosative stress and PD pathogenesis (Rizor et al., 2019). Astrocyte dysfunction can lead to impaired nutritional support and metabolic disorders of neurotransmitters, which aggravate neuronal damage and death and promote the process of neurodegeneration (Lee et al., 2022). These high-frequency keywords are the hotspots in the research topic and reflect the mainstream direction.

Keyword burst analysis can identify keywords with a significant increase in frequency in the literature at a particular time. These keywords usually represent current research hotspots and trends in the field. In recent years, NLRP3 inflammasome, EVs, and signaling pathway were the keywords with strong burst force. In the pathological process of PD, the excessive activation of NLRP3 inflammasome in astrocytes is one of the key links leading to inflammatory response (Nguyen et al., 2022). A cross-sectional study showed that serum NLRP3 levels were significantly increased and inflammatory markers were elevated in PD patients (Chatterjee et al., 2020). The dopamine D2 receptor can inhibit NLRP3 inflammasome-mediated neuroinflammation in astrocytes by enhancing the interaction between β-arrestin2 and NLRP3 (Zhu et al., 2018). Activation of cannabinoid receptor 2 on astrocytes can significantly reduce NLRP3-mediated neuroinflammation and alleviate movement disorders in PD mice (Zhu et al., 2023). EVs released by astrocytes from the nigrostriatal system have a neuroprotective effect in PD, which can rescue mitochondrial complex I function and restore ATP production in neurons damaged by the neurotoxin MPP (Leggio et al., 2022). Neuron-derived EVs can exacerbate PD by transferring cytotoxic oligomeric and multimolecular forms of α-Syn between neurons and nonneuronal cells, such as astrocytes and microglia (Chistiakov and Chistiakov, 2017). A clinical study showed that the number of astrocyte-derived EVs carrying α-Syn in the peripheral blood of PD patients was significantly higher than that of healthy controls, which may become an effective biomarker for the clinical diagnosis or differential diagnosis of PD (Wang et al., 2023). The astrocyte-mediated Wnt/β-catenin signaling cascade plays a key role in neuroprotection and self-repair, which is essential for the survival and protection of midbrain dopaminergic neurons (Marchetti et al., 2013). Transient receptor potential vanilloid 1 (TRPV1) activation on astrocytes increases ciliary neurotrophic factor (CNTF) synthesis to enhance the viability of dopaminergic neurons to exert neuroprotective effects in the TRPV1-CNTF signaling pathway (Nam et al., 2015). JWA in astrocytes can inhibit the activation of NF-kB signaling pathway and reduce neuroinflammatory response by down-regulating the expression of IKKβ (Miao et al., 2014). These research directions may be therapeutic targets for PD.

There are some limitations in this study. Data were derived from WoS and publications from other databases were not included. The analysis of publications focused on articles and reviews in English, and some publications in other languages and types may have been missed.

## 5 Conclusion

Collaboration among different countries, organizations, and authors has effectively promoted the rapid development of this field, and the research achievements have gradually increased. The research hotspots mainly focused on neuroinflammation, α-Syn, microglia, oxidative stress, and neurodegeneration. NLRP3 inflammasome, EVs, and signaling pathway are research directions in the future.

## Data Availability

The original contributions presented in the study are included in the article/supplementary material, further inquiries can be directed to the corresponding authors.
